# When Evaluating a New Thyroid Mass and a Ring-Enhancing Brain Lesion (When Two Presentations Collide)

**DOI:** 10.7759/cureus.600

**Published:** 2016-05-04

**Authors:** Zachary Nicholas, Michael Sughrue, James Battiste, Ozer Algan

**Affiliations:** 1 Department of Radiation Oncology, University of Oklahoma; 2 Department of Neurosurgery, University of Oklahoma; 3 Neurooncology, University of Oklahoma

**Keywords:** thyroid nodule, multiple sclerosis, radiation therapy, tumefactive, thyroid cancer

## Abstract

We aimed to evaluate the clinical and pathologic features of two common medical illnesses and their appropriate workup and pathognomonic findings. A 57-year-old white male presented with a new onset expressive aphasia while traveling abroad. He was evaluated at an outside facility and underwent workup for a stroke. The evaluation included a CT and MRI of the brain demonstrating three new enhancing lesions, the largest of which was a 2.5 cm ring-enhancing cystic lesion. A CT of the chest noted a 4-cm cystic thyroid lesion that was diagnosed as a thyroid cancer with brain metastases. The patient was told that he had cancer and needed therapy. The patient elected to be treated closer to home and presented to our institution with a referral for brain irradiation. The patient was evaluated and his case was reviewed in a neuro/oncology tumor board, where several other possible diagnoses were considered. A complete workup was performed, including two separate FNAs of the thyroid mass along with a PET scan, CEA test, CBC test, CMP, CRP, sed rate, and SLE testing, along with a spinal tap (cytology, protein, and serology).

The MRI on further review showed that one of the lesions was a periventricular enhancing area and the largest lesion was an open ring with T2 and DWI enhancement. The fine needle aspiration (FNA) samples of the thyroid both showed benign histology. The laboratory evaluation was negative except for a mildly elevated CRP with no tumor markers identified and the spinal tap was positive for elevated protein and particularly oligoclonal bands. The PET scan showed no sites of fluorodeoxyglucose (FDG) avid masses including the thyroid. Multiple sclerosis (MS) represents 400,000 cases in the US and benign thyroid nodules noted on imaging range from 19-35% of the population. One pathognomonic finding of MS that is less common is the open rings called tumefactive lesions versus the closed rings seen with metastases. A cystic thyroid lesion can range from a benign process to a differentiated thyroid cancer. The rate of distant metastasis with these cancers ranges from 1-23% in the literature. Lung and bone metastasis are the most common sites with CNS metastasis only accounting for < 2% of the cases. A better understanding of these findings should allow physicians to have a higher degree of suspicion in these cases and provoke further inquiry to prevent unnecessary injury.

## Introduction

This is a case report that investigates a new diagnosis of metastatic thyroid cancer to the brain. This paper illustrates important principles to be considered during workup of patients with thyroid and brain lesions on imaging. Informed consent was obtained from the patient for this study.

## Case presentation

**Figure 1:**
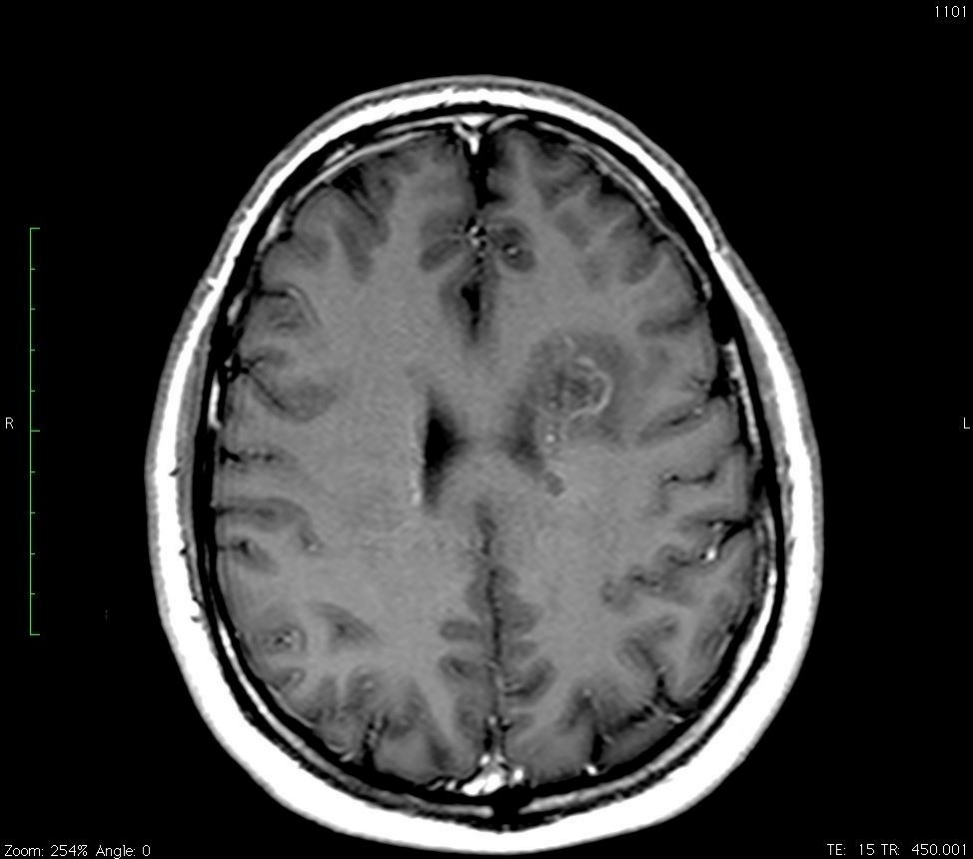
T1 MRI post contrast with a contrast-enhancing lesion with the “Open Ring” appearance.

**Figure 2:**
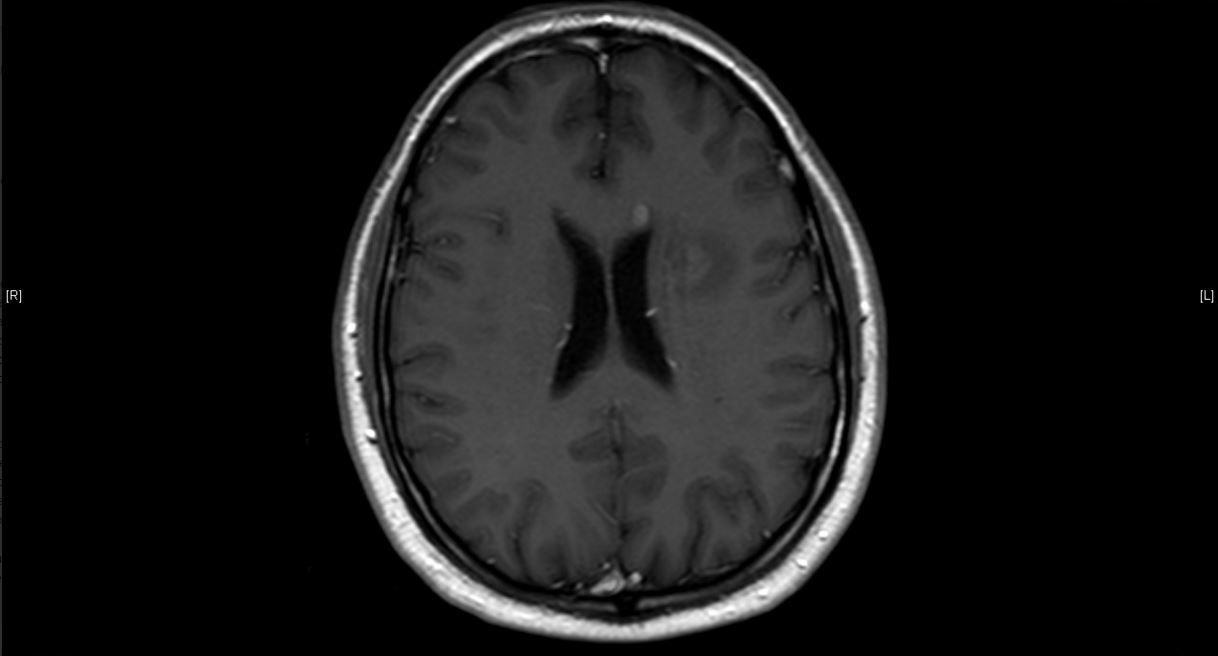
T1 with contrast MRI image showing a periventricular enhancing lesion anteriorly in the left side of the genu of the corpus callosum. The larger periventricular open ring cystic lesion is also noted in the same image.

**Figure 3:**
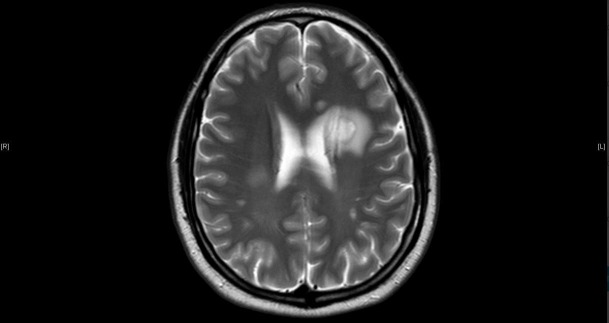
T2 MRI image showing the corresponding T2 enhancement of the aforementioned lesions.

The patient is an otherwise healthy 57-year-old male who developed a left-sided facial droop and an expressive aphasia on January 20, 2014, while on a cruise in the Caribbean. The patient was examined by the physician on the cruise and given aspirin with the initial concern of a potential stroke. The next day he was seen in Jamaica and then transferred to Florida with further concerns for a stroke. A computed tomography (CT) scan of the head did not reveal an acute hemorrhage but had findings that were concerning for an intracranial mass. A subsequent magnetic resonance imaging (MRI) of the brain (Figures 1-3) revealed three separate contrast-enhancing nodules, one < 1 cm in the left periventricular region, a second, 2.5 cm in size in the left periventricular/parietal region with a hemorrhagic core, and a third nodule, 1.5 cm in size in the right parietal region.

The two larger lesions both exhibited significant surrounding T2 and DWI enhancement. Some smaller T2 intense foci were noted as well but were felt to be indeterminate in nature. These lesions appeared to represent brain metastases from the outside medical center's report. An initial workup for a primary disease site included a CT scan of the chest/abdomen/pelvis, which only revealed a partially visualized left thyroid nodule. A CT scan of the neck revealed a 4-cm, well-circumscribed left thyroid mass that was heterogeneous with a fluid core along with several areas of septation. The mass was further evaluated via ultrasound (US) that noted a 3.5 cm left thyroid mass with a fluid-filled core with septation. The patient was then informed that he had thyroid cancer with brain metastases at the outside center and was told he needed brain radiation therapy. The patient was started on 2 mg dexamethasone twice daily and he opted to return home for further care.

**Figure 4:**
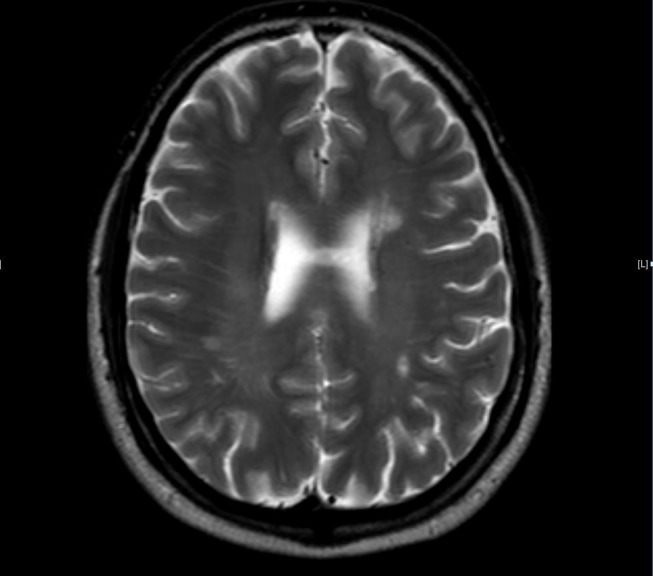
T2 MRI follow-up imaging obtained 17 months after his diagnosis of multiple sclerosis.

When the patient was first assessed, he appeared to be stable. After starting the steroids, he noted some improvement in his facial droop and expressive aphasia. The patient was set up for ears, nose, and throat (ENT) evaluation, and his outside images were discussed in a neuro-oncology conference. Review of his brain images did raise the possibility of brain metastases, although another diagnosis, such as central nervous system (CNS) lymphoma or a demyelinated process, were also discussed, and further workup was initiated. The patient underwent a positron emission tomography (PET) scan that noted the isointense left thyroid lesion and two isointense brain lesions but no sites of hypermetabolism. His thyroid stimulating hormone (TSH) was 0.5 mIU/ml (nml 0.35-4.94), T4 was 5.4 mcg/dl (nml 4.1-11.0) and T3 uptake was mildly elevated at 42%. Initial thyroid fine needle aspiration (FNA) procedure on January 27 revealed no malignant cells, although it appeared to be indeterminant in nature. Repeat US-guided FNA procedure on January 29 also revealed no malignant cells with adequate specimen obtained this time. His comprehensive metabolic panel (CMP) was noncontributory. Lactate dehydrogenase (LDH) was 109 U/L (nml 90-190). Alanine aminotransferase/aspartate aminotransferase (ALT/AST) were normal. His C-reactive protein (CRP) was 0.4. The remainder of the other tumor markers evaluated was within normal limits. The patient underwent a lumbar puncture that was negative for malignant cells and cultures but had an elevated cerebrospinal fluid (CSF) IgG at 13.7, CSF albumin to serum index at 17, IgG index at 0.8 and myelin basic protein at 2.3. Six oligoclonal bands were noted only in the CSF and the CSF HTLV-I/II antibody test was negative. At this point in time, he was given a final diagnosis of multiple sclerosis (MS) with a benign thyroid nodule. The patient was followed-up with serial imaging of the brain; due to the location of the lesions, the benign thyroid pathology and the diagnosis of MS no invasive biopsies were obtained. Subsequent MRI imaging coincided with the diagnosis of multiple sclerosis (Figure 4).

## Discussion

The frequency of distant metastasis on initial presentation of differentiated thyroid cancer ranges from 1% to 23% [1-2]. Follicular and Hurthle cell represent higher rates of distant metastasis than papillary histology. Undifferentiated or anaplastic thyroid carcinoma carry higher rates of brain metastasis but are less likely to be confused with a benign cystic thyroid lesion at initial presentation. In patients that develop metastasis from primary differentiated thyroid cancer, lung predominates at 45% with the second being bone at 39%. Brain metastasis represents only 0.15-2% of the differentiated thyroid cancers at presentation [1, 3-4]. Of the patients that present with thyroid nodules, only five percent are found to be malignant [5]. With the low rates of an incidental thyroid nodule representing actual malignancy and the even lower rates of brain metastasis from newly diagnosed differentiated thyroid cancer, the expected rate of these lesions representing brain metastasis is roughly 0.05%. The ultrasound-guided biopsy was the most helpful with two samples being negative for malignancy and adequate specimen sampling noted with the second.

Thyroid cancer metastasis, lymphoma, and demyelinating syndromes were the primary differential for the brain lesions initially. Contrast-enhancing brain lesions have a large differential diagnosis but with an extracranial suspicious mass, malignancy rises quickly in the differential. Tumefactive multiple sclerosis is in the differential for intracranial ring-enhancing lesions as well. This is a disease process that represents one to two out of every 1000 cases of MS in the U.S. [6]. MS represents 400,000 cases in the US and two million cases worldwide [7]. Multiple sclerosis is characterized by the periventricular lesions that were noted in this patient’s case. The necrotic core noted in one of the lesions in this patient made an autoimmune disease process less probable in the initial workup. Subsequently the presence of the elevated IgG and the classic oligoclonal bands noted in the CSF specimen increased the likelihood that MS was the primary cause for these brain lesions. The remainder of the workup was essential to rule out any other potential sites of malignant disease. The key step in his diagnosis was explaining the presentation of the two enlarged enhancing necrotic lesions. The presentation of tumefactive MS has been reported to mimic a stroke-like presentation that is consistent with the initial presentation in this case [8]. The imaging findings, in this case, are also consistent with the text description of this process with contrast-enhancing rings with T2 enhancement as well in the surrounding tissue. Tumefactive lesions tend to be larger than 2 cm on presentation and are noted to have an “open ring” vs a complete ring that can be seen in an abscess or tumor. The open ring has been shown to have a likelihood ratio of representing demyelinating syndromes versus neoplasms of 5.2 and 17.2 versus infection [9]. The specificity of calling a lesion based on the open ring by a neuroradiologist has been reported at 93.8% [9]. The concerning lesion of interest in this study was 2.5 cm in size and had an open ring appearance consistent with other case reports, the laboratory values were diagnostic for MS, the thyroid and malignant workup was negative, and ultimately, this evidence was convincing that the diagnosis was multiple sclerosis and a benign thyroid nodule and therapy could be initiated for MS [9-10].

## Conclusions

This case represents a single case report of a patient with a newly diagnosed tumefactive multiple sclerosis and a benign thyroid nodule that was evaluated to rule out malignancy prior to further therapy for an autoimmune process. This presentation illustrates the paradigm that thyroid lesions in particular represent a relatively low risk of malignancy and metastasis to the brain. The case also illustrates the need to confirm outside reports and diagnosis as this patient came to a specialist’s clinic with a request from an outside clinic for brain radiation therapy for metastatic cancer. We conclude that autoimmune disease can be confused with a malignant presentation and referred the patient to an oncologist for workup, which requires a continued use of a differential diagnosis that may include non-malignant conditions. 
